# Prevalence of asthma–COPD overlap syndrome among primary care asthmatics with a smoking history: a cross-sectional study

**DOI:** 10.1038/npjpcrm.2015.47

**Published:** 2015-07-16

**Authors:** Toni Kiljander, Timo Helin, Kari Venho, Antero Jaakkola, Lauri Lehtimäki

**Affiliations:** 1 Department of Respiratory Diseases, Terveystalo Hospital, Turku, Finland; 2 Department of Allergology, Helsinki University Central Hospital, Helsinki, Finland; 3 Department of Respiratory Medicine, Central Hospital of Central Finland, Jyväskylä, Finland; 4 Boehringer Ingelheim Finland, Helsinki, Finland; 5 Department of Respiratory Medicine, University of Tampere, Tampere, Finland

## Abstract

**Background:**

The overlap between asthma and chronic obstructive pulmonary disease (COPD) is an important clinical phenomenon. However, the prevalence of asthma–COPD overlap syndrome (ACOS) is not known.

**Aims:**

To investigate the prevalence of ACOS among asthmatic patients with a smoking history, and evaluate the factors predicting ACOS in this patient group.

**Methods:**

We investigated 190 primary care asthma patients with no previous diagnosis of COPD, but who were either current or ex-smokers, with a smoking history of at least 10 pack-years. Spirometry was performed on all the patients while they were taking their normal asthma medication. Patients were considered to have ACOS if their postbronchodilator forced expiratory volume in 1 s/forced vital capacity was <0.70.

**Results:**

Fifty-two (27.4%) of the patients were found to have ACOS. Age ⩾60 years and smoking for ⩾20 pack-years were the best predictors of ACOS. If both of these criteria were met, the odds ratio (95% confidence interval) for ACOS was 6.08 (2.11–17.49), compared with the situation where neither of these criteria were fulfilled.

**Conclusions:**

There is a high prevalence of ACOS among primary health care asthmatics with a positive smoking history but no previous diagnosis of COPD. In this population, age over 60 years and a smoking history of more than 20 pack-years were the best predictors of ACOS.

## Introduction

Asthma and chronic obstructive pulmonary disease (COPD) are the two most common obstructive pulmonary diseases. Although asthma and COPD most often represent two distinct diseases, there is also significant overlap between these two diseases.^1,2^

The definition of asthma–COPD overlap syndrome (ACOS) is undetermined. Most commonly, it is defined as either the diagnosis of COPD in a patient with previously diagnosed asthma, or as incompletely reversible airway obstruction accompanied by symptoms or signals of increased reversibility of the obstruction.^3^ A recent update of the GINA report recommended a stepwise approach to the diagnosis of ACOS, and defined it as a syndrome characterized by persistent airflow limitation with several features usually associated with asthma and several features usually associated with COPD.^1^

Compared with asthma or COPD alone, ACOS is associated with worse health-related quality of life,^4,5^ more frequent exacerbations,^5,6^ increased hospitalisation^6,7^ and higher health care costs.^8^ Although ACOS appears to be clinically highly significant, little is known about the treatment of these patients, as they are typically excluded from therapy trials for asthma or COPD.^9^

There is only some data on the prevalence of ACOS. Hardin *et al.*^5^ found that 13% of the COPD patients in the COPDGene study reported a history of doctor-diagnosed asthma. Similarly, Miravitlles *et al.*^10^ reported that 17.4% of COPD patients in the EPI-SCAN study reported that they had been previously diagnosed with asthma. The prevalence of overlap syndrome has been shown to increase with age,^11^ which may reflect the fact that, over the years, asthmatics may develop fixed airway obstruction, especially if they do not use anti-inflammatory medication^12^ or if they smoke.^13^ Soriano *et al.*^11^ found that as many as half of the patients with obstructive pulmonary disease, aged 50 years or more, had simultaneously more than one obstructive condition.

The aim of the present study was to investigate the prevalence of undiagnosed ACOS among primary health care asthma patients who are current or ex-smokers, and the factors predicting ACOS in this patient group.

## Materials and methods

This was a cross-sectional study. Patients were recruited mostly from the appointments of primary care physicians and through newspaper advertisements. In addition, a few patients were also recruited by private pulmonologists treating primary care-like asthma patients.

The study was approved by the Ethics Committee of Pirkanmaa Health Care District, and every patient gave written informed consent before any study-related procedures were performed.

### Patients

The inclusion criteria were as follows: age 18–70 years, current or ex-smoker with 10 or more pack-years, doctor-diagnosed asthma with special reimbursement for asthma medication granted by the National Health Insurance. To qualify for this reimbursement, patients must have fulfilled at least one of the following criteria: (1)⩾12% (and 200 ml) reversibility in forced expiratory volume in 1 s (FEV_1_) or forced vital capacity (FVC) in a bronchodilation test, (2) during a 2-week peak expiratory flow monitoring at least three times either a bronchodilator response of ⩾15% (and 60 l/min) or a diurnal variation of ⩾20%, (3) moderate-to-severe bronchial hyperresponsiveness in histamine or methacholine inhalation challenge or (4) FEV_1_ had improved more than 15% during a corticosteroid treatment test.

The exclusion criteria were as follows: any severe illness, any known pulmonary disease other than asthma, use of inhaled anticholinergic or indacaterol or oral roflumilast.

### Methods

After informed consent was received, the investigator and the patient completed a questionnaire including questions about the inclusion and exclusion criteria, and the patient’s asthma medication, symptoms and exacerbations. The investigator stored the data in a database. Body mass index and current smoking status were recorded as a standard procedure when spirometry was performed.

Spirometries (Medikro Kuopio, Finland) were performed according to the guidelines^14^ before and after administration of 400 μg of inhaled salbutamol while the patients were taking their usual asthma medication.

The patients were considered to have ACOS if their postbronchodilator FEV_1_/FVC was less than 0.70. Patients with ACOS were divided into GOLD grades of airway obstruction according to the GOLD report.^2^

Patients were considered to have had an exacerbation during the previous year if they had been hospitalised, or had used a course of oral corticosteroids, for their asthma during the previous year.

### Statistical analysis

The primary outcome was the prevalence of ACOS, as defined above, among asthmatics with a smoking history of at least 10 pack-years. The sample size calculation was based on the assumption that the prevalence of ACOS in asthmatics is approximately 45%.^11^ Using the large sample normal approximation, 265, 195, 149, 118 or 96 patients would be required to estimate the prevalence to be 95% confident that the estimate will not differ from the true prevalence by more than 6, 7, 8, 9 or 10 percent, respectively. The final sample size of 219 recruited patients was assessed to be large enough to obtain a sufficient precision. The 95% confidence interval for prevalence was calculated using the large sample normal approximation. The distribution of the variables was checked using the Kolmogorov–Smirnov test and graphical plots. The distributions of demographic continuous data were skewed and are expressed as medians (interquartile range). The nonparametric Mann–Whitney *U*-test was used to compare the groups with and without ACOS with respect to continuous variables. The Chi-square test and the exact Fisher's test, when appropriate, were used for categorical variables. Spearman’s rank correlation (Rho) was used to study the associations between postbronchodilator FEV_1_/FVC versus age and pack-years. The receiver operating curve analysis (ROC) was used to determine the best cut-off values of age and pack-years to differentiate between asthma patients with and without overlap syndrome. Sensitivity and specificity were assessed to be equally important when the best cut-off values were chosen. In addition, positive predictive values and negative predictive values were calculated. The potential prognostic factors for overlap were sex, age, body mass index, current smoking status and pack-years of smoking. Univariable logistic regression analyses were performed to study the associations. The results are given as odds ratios with 95% confidence intervals. *P* values less than 0.05 were considered statistically significant. The analyses were performed using IBM SPSS Statistics for Windows (version 22.0, Armonk, NY, USA, IBM Corp.).

## Results

**Figure 1:**
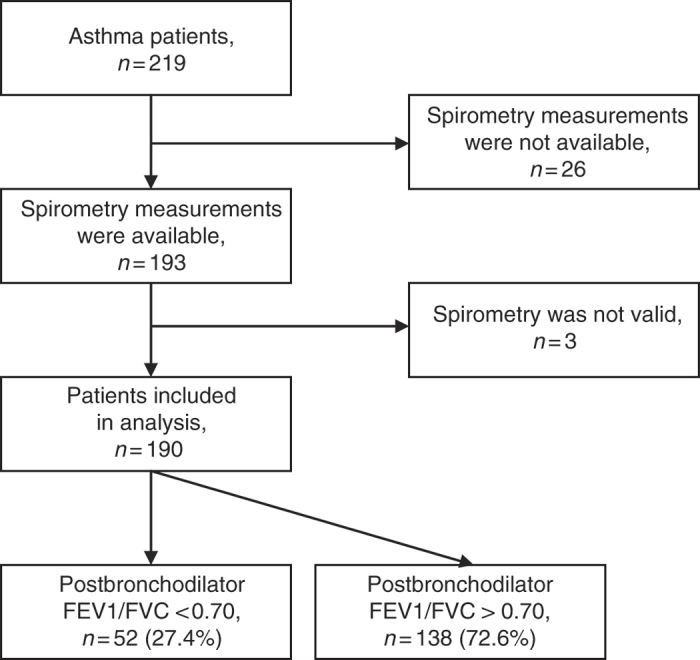
Flow of participants. FEV_1_, forced expiratory volume in 1 s; FVC, forced vital capacity.

Two hundred and nineteen patients were recruited and 190 of them were included in the analysis (Figure 1). Their median age (range) was 58 (23–70) years, they had smoked for 20 (10–60) pack-years, and their body mass index was 27.5 (16.1–50.3) kg/m^2^. Eighty-three (44.1%) of the patients were current smokers and 112 (58.9%) were female.

Fifty-two (27.4%, 95% confidence interval 21–34%) patients were found to have postbronchodilator FEV_1_/FVC <0.70 and were thus considered to have ACOS. Twelve (23.1%), 38 (73.1%) and 2 (3.8%) belonged to GOLD stages 1, 2 and 3, respectively. None of the patients belonged to GOLD stage 4.

A comparison of the patients with ACOS and those with asthma alone is shown in Table 1. Patients with overlap syndrome were older and had smoked more than patients with asthma alone. Overlap patients tended to be more often current smokers and to have more often significant reversibility, but these differences were not statistically significant.

**Figure 2:**
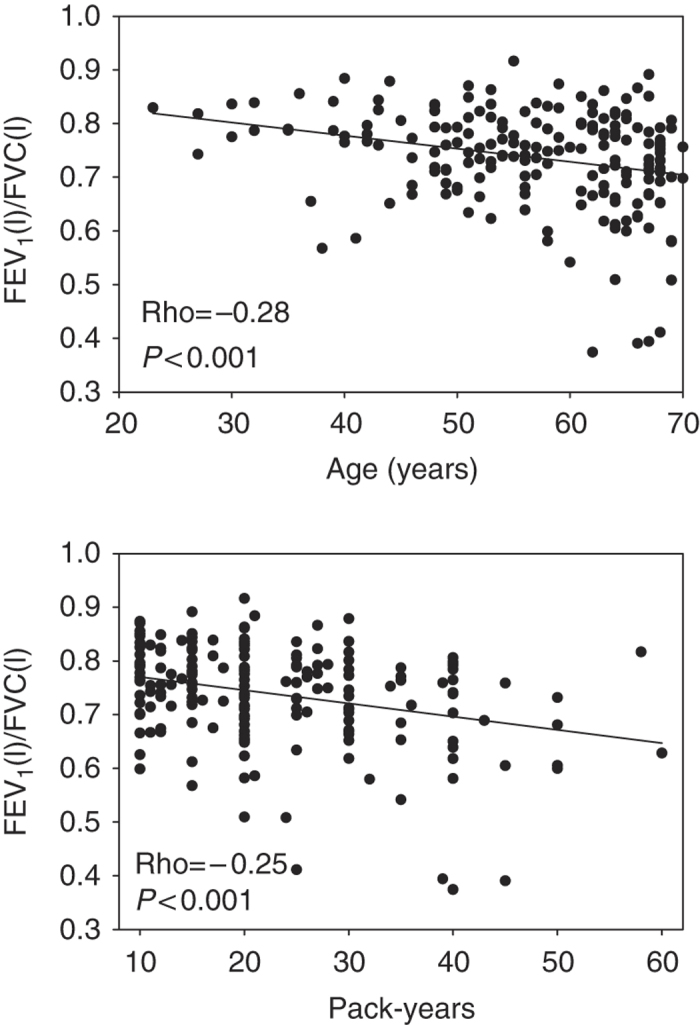
Scatter plots and regression lines showing the association between age and pack-years versus postbronchodilator FEV_1_/FVC in 190 asthma patients with a positive smoking history. FEV_1_, forced expiratory volume in 1 s; FVC, forced vital capacity.

A negative correlation between postbronchodilator FEV_1_/FVC and age (Spearman Rho=−0.28, *P*<0.001) and pack-years (Rho=−0.25, *P*<0.001) was found (Figure 2).

**Figure 3:**
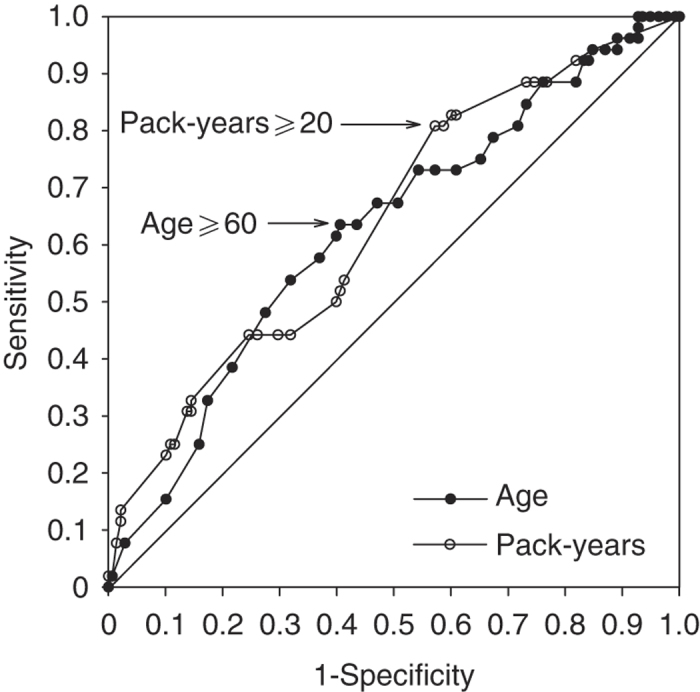
Receiver operating curve (ROC) analysis of age and pack-years in 190 asthmatic patients. The cut-off points of 60 years of age yielded 63.5% sensitivity and 59.4% specificity and the cut-off point of 20 pack-years yielded 80.8% sensitivity and 42.8% specificity to detect overlap syndrome.

ROC analysis for age and pack-years revealed that age ⩾60 years and smoking for ⩾20 pack-years were the best predictors of ACOS in the study population (Figure 3). The area under the ROC curve was 0.625 and 0.639 for age and pack-years, respectively. The cut-off point of 60 years of age yielded 63.5% sensitivity and 59.4% specificity, and the cut-off point of 20 pack-years 80.8% sensitivity and 42.8% specificity, to detect overlap syndrome (Table 2). However, the combination of both age ⩾60 years and smoking for ⩾20 pack-years yielded the best area under the ROC curve: 0.670, and 53.8% sensitivity and 74.6% specificity.

The results of the logistic regression analysis are shown in Table 3. Using the cut-off points given by the ROC analysis, age and pack-years were the only significant factors predicting overlap syndrome. If the patient was at least 60 years old and had simultaneously smoked for at least 20 pack-years, the odds ratio (95% confidence interval) for overlap syndrome was 6.08 (2.11–17.49), *P*=0.001, compared with the situation where neither of these criteria were fulfilled. In patients with one criterion fulfilled (age ⩾60 or smoking for ⩾20 pack-years), the risk of ACOS was about twice as high as for patients who were younger than 60 years and had smoked less than 20 pack-years. In other words, if neither of the criteria (age ⩾60 years and smoking ⩾20 pack-years) were fulfilled, the prevalence of overlap syndrome was 11.6% (5/43 patients). If one of the criteria was met, then overlap syndrome was found in 22.6% (19/84) of the patients. If both criteria were met the prevalence of overlap syndrome was found to be 44.4% (28/63 patients).

## Discussion

### Main findings

We found the prevalence of asthma–COPD overlap syndrome to be 27.4% among primary health care asthmatics with no previous diagnosis of COPD, but who were either current or ex-smokers with a smoking history of at least 10 pack-years. The patients with ACOS were older and had smoked more than patients with asthma alone.

### Strengths and limitations of this study

The current study has its weaknesses. First, the number of participants was relatively small to investigate the differences between patients with ACOS and asthma alone. However, we were able to find that older age and heavier smoking history predict ACOS, which are the most common variables found in other studies too. Moreover, with 190 participants, we were able to evaluate the prevalence of ACOS among asthmatics with a positive smoking history, which was the primary objective of the study. Second, as we did not investigate consecutive patients, there might have been selection bias. On the other hand, as we excluded patients with known COPD, and even those using the drugs most commonly prescribed for COPD, one could speculate that the result might rather be biased the other way. Third, as a few patients were recruited by private pulmonologists, one could argue that we did not investigate exclusively primary care patients. However, in Finland, treatment of the most severe asthmatics is concentrated in central and university hospitals, and the recruiting private pulmonologists were emphasised to recruit only patients who could also be treated by general practitioners. Moreover, as ACOS patients’ COPD was mostly mild, with 96% being classed as GOLD stages 1 and 2, we strongly believe that we investigated primary health care outpatients as was intended.

The study also has its strengths. First, as the criteria for special reimbursement for asthma medication are strict, we are positive that all the patients investigated really had asthma. Second, as far as we know this is the first study to investigate the prevalence of ACOS among primary health care asthmatics with a positive smoking history but without a previous diagnosis of COPD. In most earlier studies, the prevalence of ACOS has been evaluated among COPD patients by asking them if they have doctor-diagnosed asthma.^5,10,15^

### Interpretation of findings in relation to previously published work

Hardin *et al.*^5^ found the prevalence of asthma–COPD overlap to be 13% among COPD patients in the COPDGene population. Miravitlles *et al.*^10^ found that 17.4% of COPD patients in the EPI-SCAN population reported they had previously been diagnosed with asthma, and thus presented with asthma–COPD overlap. In the PLATINO study, 22.8% of patients with FEV_1_/FVC <0.7 reported a prior diagnosis of asthma and can be considered as overlap patients.^15^ The 27.4% prevalence of asthma–COPD overlap syndrome found in our study is slightly higher than in previous studies. We believe that the major reason for this difference may be the fact that we investigated a different patient population. We studied asthmatic patients and investigated how often their postbronchodilator FEV_1_/FVC is <0.7, whereas other studies^5,10,15^ have investigated how often patients with postbronchodilator FEV_1_/FVC <0.7 report that they have previously been diagnosed with asthma. On the other hand, prevalences of overlap even higher than ours have been suggested. For example, Soriano *et al.*^11^ found that as many as 50% of patients with obstructive pulmonary disease, aged 50 years or more, may suffer simultaneously from more than one obstructive condition.

The high prevalence of ACOS may be explained by the fact that asthma and airway hyperresponsiveness are suggested to be risk factors for developing COPD.^2^ Lange *et al.*^16^ have shown that decline in pulmonary function is faster in asthmatics than among those without asthma, and that the decline is fastest among those asthmatics who smoke. In the study by Vonk *et al.,*^12^ 16% of patients with asthma developed irreversible airway obstruction during a 26-year follow-up. It has been shown that airway hyperresponsiveness, even without asthma, is an important risk factor for developing COPD.^17^

Smoking is the most important risk factor for COPD.^2^ Therefore, it is not surprising that the ACOS patients in our study had smoked more than those with asthma alone. Also in the study by Lee *et al.,*^18^ greater amount of cigarette smoking was related to the development of fixed airway obstruction among asthmatic patients.

We found ACOS patients to be older than patients with asthma alone. In the study by Menezes *et al.,*^6^ patients with asthma–COPD overlap were also older than patients with asthma alone. In that study, and in the COPDGene study,^5^ patients with COPD were older than the overlap patients. These findings might suggest a continuum from reversible airway obstruction, via overlap, to irreversible obstruction in some patients. There is evidence that some patients with asthma developed irreversible airway obstruction during long-enough follow-up,^12^ and that longer duration of asthma may be associated with irreversible airway obstruction.^18^ Unfortunately, in the current study, we were unaware of how long the patients had had asthma. However, we believe that the age of the patients indirectly reflects the duration of asthma, and therefore, our finding that age is associated with low FEV_1_/FVC is in keeping with the results of the studies suggesting that longer duration of asthma may be associated with irreversible airway obstruction. On the other hand, aging *per se* causes changes in lung elastic recoil and pulmonary mechanics that causes FEV_1_/FVC to decrease; hence, older subjects may be more prone to fulfil the diagnostic criteria of ACOS regardless of the duration of their asthma.^19^

### Implications for future research, policy and practice

In the current study, the best predictors of ACOS were smoking for ⩾20 pack-years and age ⩾60 years. If both of these criteria were met, then ACOS was found in almost half of the patients. Lee *et al.*^18^ found that longer duration of asthma and greater amount of cigarette smoking were associated with fixed airway obstruction, that is, ACOS, in patients with severe asthma. In everyday life, this could mean that in the case of an elderly asthmatic with a clearly positive smoking history, whose asthma is difficult to treat, attention should be given not just to the known asthma, but also one should take the COPD component of possible ACOS into account as well.

### Conclusions

There is a high prevalence of ACOS among asthmatics with a positive smoking history but no previous diagnosis of COPD. Age over 60 years and smoking for more than 20 pack-years were the best predictors of ACOS in this population.

## Figures and Tables

**Table 1:** Characteristics of 190 asthmatics with and without overlap syndrome

	*Patients with overlap syndrome* *(*N*=52)*	*Patients with asthma only* *(*N*=138*)	P *value*^a^
Age (years)	63.0 (52.5–66.5)	57.0 (49.0–64.0)	0.008
Pack-years	24.5 (20.0–37.0)	20.0 (13.0–28.0)	0.003
BMI (kg/m^2^)	26.2 (23.2–29.9)	27.8 (24.6–31.6)	0.09
Current smokers	28 (54.9%)	55 (40.1%)	0.07
Females	31 (59.6%)	81 (58.7%)	0.91
Inhaled corticosteroid (ICS)	50 (96.2%)	129 (93.5%)	0.48
Inhaled corticosteroid+inhaled long-acting β_2_-agonist (ICS+LABA)	36 (69.2%)	86 (62.3%)	0.38
Short-acting β-agonist (SABA) more often than twice a week	19 (36.5%)	37 (26.8%)	0.19
Exacerbation during previous year	16 (30.8%)	35 (25.4%)	0.45
Significant reversibility	8 (15.4%)	9 (6.5%)	0.08^b^

All patients were current or ex-smokers. Results are given as median (interquartile range) or number (%).

Abbreviation: BMI, body mass index.

a Mann–Whitney *U*-test was used for continuous variables and Chi-squared test for categorical variables.

b Fisher's exact test.

**Table 2:** The best cut-off values of age and pack-years and their combination to detect asthma–COPD overlap syndrome among 190 asthmatics with positive smoking history

	*Sensitivity* *(%)*	*Specificity* *(%)*	*PPV* *(%)*	*NPV* *(%)*
Age ⩾60 years	63.5	59.4	37.1	81.2
Pack-years ⩾20	80.8	42.8	34.7	85.5
Age ⩾60 years or pack-years ⩾20	90.4	27.5	32.0	88.4
Age ⩾60 years and pack-years ⩾20	53.8	74.6	44.4	81.1

Abbreviations: COPD, chronic obstructive pulmonary disease; NPV, negative predictive value; PPV, positive predictive value.

**Table 3:** Asthma–COPD overlap syndrome in association to demographic characteristics in 190 asthma patients with positive smoking history. Results are given by univariable binary logistic regression analyses

	*Overlap syndrome*		*Unadjusted*		P *value*
	N^a^	*(%)*	*OR*	*95% CI*	
*Sex*					
Female	31/112	(27.7)	1.00		
Male	21/78	(26.9)	0.96	0.50–1.84	0.91
					
*Smoking*					
Ex-smoker	23/105	(21.9)	1.00		
Current smoker	28/83	(33.7)	1.82	0.95–3.47	0.07
					
*BMI, kg/m*^*2*^					
<25.0	18/56	(32.1)	1.00		
25.0–29.9	21/72	(29.2)	0.87	0.41–1.85	0.72
⩾30.0	12/60	(20.0)	0.53	0.23–1.23	0.14
					
*Age, years*					
20–59	19/101	(18.8)	1.00		
60–70	33/89	(37.1)	2.54	1.32–4.91	0.005
					
*Pack-years*					
10–19	10/69	(14.5)	1.00		
20–60	42/121	(34.7)	3.14	1.46–6.76	0.004
					
*Age and pack-years*					
Age <60 and pack-years <20	5/43	(11.6)	1.00		
Age ⩾60 or pack-years ⩾20	19/84	(22.6)	2.22	0.77–6.43	0.14
Age ⩾60 and pack-years ⩾20	28/63	(44.4)	6.08	2.11–17.49	0.001

Abbreviations: CI, confidence interval; COPD, chronic obstructive pulmonary disease; OR, odds ratio.

a Number of patients with overlap syndrome/number of all patients in the group.

## Notes

TK received personal fees from Boehringer Ingelheim Finland, GSK, Novartis and Mundipharma. TH received personal fees from Boehringer Ingelheim Finland and Takeda. KV received personal fees from Boehringer Ingelheim Finland, Chiesi, GSK, Novartis, Takeda, Teva, Mundipharma and Almirall. AJ is an employee of Boehringer Ingelheim Finland. LL received personal fees from Almirall, Astra-Zeneca, Boehringer Ingelheim Finland, Chiesi, GSK, Novartis, Orion Pharma, Takeda and Mundipharma.
